# Diversity of ThDP‐Dependent Enzymes Forming Chiral Tertiary Alcohols

**DOI:** 10.1002/cbic.202500200

**Published:** 2025-05-27

**Authors:** Daniela Bjarnesen, Lucrezia Lanza, Francesco Presini, Pier Paolo Giovannini, Michael Müller

**Affiliations:** ^1^ Institute of Pharmaceutical Sciences University of Freiburg Albertstrasse 25 79104 Freiburg Germany; ^2^ Department of Chemical Pharmaceutical and Agricultural Sciences University of Ferrara Via L. Borsari 46 44121 Ferrara Italy

**Keywords:** α-hydroxy ketones, benzoin condensations, biocatalysis, carboligations, ThDPs

## Abstract

Thiamine diphosphate (ThDP)‐dependent enzymes are well known biocatalysts for C—C bond–forming reactions. While this enzyme class is mainly investigated for the formation of acyloins of secondary alcohols, recent studies have expanded its scope to utilize ketones as electrophiles in asymmetric carboligation reactions for the formation of tertiary alcohols. Chiral tertiary alcohols are ubiquitous motifs in natural products and important building blocks for the synthesis of bioactive compounds. ThDP‐dependent enzymes are emerging as one of the most promising classes of biocatalysts for synthesizing a wide range of products due to the variety of possible substrate combinations, accessible starting materials, high enantioselectivity, and advantageous self‐regeneration of the catalytic ThDP cofactor. This review provides an overview of the ThDP‐dependent enzymes (e.g., decarboxylase, DC; transketolase, TK; α‐keto acid dehydrogenase 2, αKADH2) that form tertiary alcohols, focusing on the substrate scope and diversity of physiological functions. The available toolbox and the characterized reactions shall serve as a starting point for future studies. Inspired by nature, an even broader diversity of classes and substrate specificities is expected in this field.

## Graphical abstract

Chiral tertiary alcohols are widespread in nature and bioactive compounds; however, their asymmetric synthesis faces several challenges. This review outlines the toolbox of ThDP‐dependent enzymes able to produce tertiary alcohols through the formation of C—C bond formation using ketones as acceptor substrates. Herein, the substrate scope, the diversity of physiological functions, and future perspectives are emphasized.

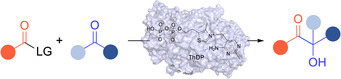

## 1 Introduction

Chiral tertiary alcohols are widespread moieties in the structures of natural products and bioactive compounds, and, as such, are important building blocks for synthesis.^[^ ^1^ ^]^ These versatile compounds can be used as precursors for further modifications, resulting in a plethora of various products. Accordingly, many different methods have been introduced for the stereoselective synthesis of tertiary alcohols.^[^ ^2^ ^]^

Nonenzymatic enantioselective methods for the synthesis of optically active tertiary alcohols have been widely investigated. Prominent strategies include hydroxylation,^[^ ^3^ ^]^ desymmetrization of prochiral tertiary alcohols,^[^ ^4^ ^]^ dihydroxylation of alkenes,^[^ ^5^ ^]^ ring‐opening of epoxides,^[^ ^6^ ^]^ kinetic resolution,^[^ ^7^ ^]^ and addition of organometallic species to ketones.^[^ ^8^ ^]^ Still, major challenges are enantioselectivity, the use of precious reagents and protecting groups, toxicity, complex reaction conditions, and the maximum theoretical yield of 50% via kinetic resolution.^[^ ^9^ ^]^ The catalytic asymmetric addition of carbon nucleophiles to ketones through organometallic reagents is the preferred synthetic process for the construction of carbon skeletons with a tertiary alcohol moiety.^[^ ^10^ ^]^ However, the reaction is synthetically demanding, because of the complex chiral auxiliaries that are required and the difficult differentiation between the two enantiotopic faces of the ketone. Despite the great progress and numerous efficient protocols described in the literature,^[^ ^2^, ^8^, ^11^ ^]^ the nonenzymatic synthesis of tertiary alcohols and their stereocontrol remain challenging.^[^ ^12^ ^]^

Alternative synthetic routes can be found in nature. Tertiary alcohol motifs are abundant in natural products such as steroids, antimicrobials, terpenoids, and structural components. Therefore, the study of biosynthetic pathways can reveal interesting enzymatic routes that serve as inspiration for biocatalysis. Commonly known enzymatic synthesis of tertiary alcohols^[^ ^13^ ^]^ include hydrolases,^[^ ^14^, ^15^, ^16^, ^17^, ^18^, ^19^ ^]^ hydratases,^[^ ^20^, ^21^, ^22^, ^23^, ^24^, ^25^, ^26^ ^]^ or oxygenases.^[^ ^27^, ^28^, ^29^ ^]^ Hydrolases catalyze epoxide ring openings or racemic resolution of tertiary alcohols, while oxygenases and hydratases create a new quaternary center by the addition of oxygen to the substrate. Several of these enzymatic reactions are highly specialized, resulting in a limited substrate scope and lower enantioselectivity toward nonphysiological substrates. Furthermore, the use of expensive cofactors limits their wide applicability.

**Scheme 1:**
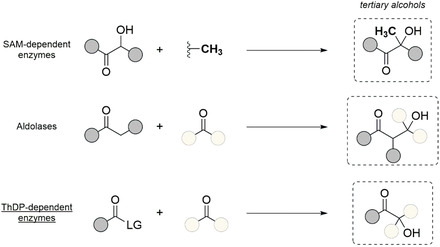
Biocatalytic carboligation methods for the synthesis of chiral tertiary alcohols.

The formation of a new quaternary center via carboligation reaction is widespread in nature and is a common method for the enzymatic production of tertiary alcohols. *S*‐adenosylmethionine (SAM)‐dependent enzymes, aldolases, and thiamine diphosphate (ThDP)‐dependent enzymes are the main enzymes known to catalyze such reactions (**Scheme** **1**).

SAM‐dependent enzymes can catalyze the methylation of the carbinol carbon of a secondary alcohol next to a carbonyl. Avilamycin, erythromycin, and cyanosporasides are some examples of natural products synthesized by these enzymes.^[^ ^30^, ^31^, ^32^ ^]^ Aldolases produce tertiary β‐hydroxy ketones through the carboligation of an enol donor and an activated ketone acceptor, such as α‐keto acids.^[^ ^33^, ^34^ ^]^ Recently also nonactivated ketones have been recognized as aldolase's acceptor substrates. The mechanism for the synthesis of tertiary alcohols has been elucidated, and further research is underway to broaden the now limited acceptance of such substrates.^[^ ^35^, ^36^ ^]^

**Scheme 2:**
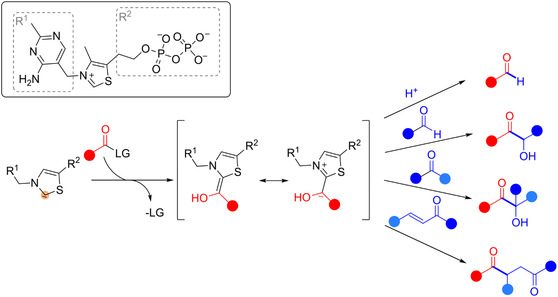
Schematic view of the ThDP cofactor and the general reaction mechanism. A donor substrate (in red) is bound to form the Breslow intermediate. Depending on the acceptor substrate, several products are accessible.

ThDP‐dependent enzymes are a large class of enzymes divided into 9 superfamilies based on structural and sequence similarity.^[^ ^37^ ^]^ The class is highly diverse and includes ligases, lyases, oxidoreductases, and transferases. Interestingly, their catalytic and functional promiscuity depends on the surrounding environment that determines their reaction specificity, although all these enzymes have the same ThDP cofactor. The catalytic mechanism starts with the attack of the cofactor on the donor substrate (**Scheme** **2**, in red) to form the Breslow intermediate. This leads to the formation of the activated (*umpoled*) aldehyde, which then can be transferred to various acceptor substrates (Scheme 2, in blue), resulting in a plethora of different products.^[^ ^38^, ^39^ ^]^ As a well‐studied class of enzymes, numerous examples of enantioselective reactions can be found in the literature in which chiral acyloins of secondary alcohols are formed in good to high yields.^[^ ^40^, ^41^ ^]^ Furthermore, the control of enantioselectivity and the broadening of the substrate scope through enzyme engineering have also already been investigated and are readily accessible.^[^ ^42^ ^]^

While ThDP‐dependent enzymes are mostly used and studied for the production of α‐hydroxy ketones of secondary alcohols (benzoin‐type reactions) and 1,4 diketones (Stetter‐type reactions),^[^ ^43^ ^]^ it is still neglected that they have an impressive potential for the production of tertiary alcohols. In particular, some ThDP‐dependent enzymes can catalyze the synthesis of tertiary α‐hydroxy ketones by addition of an acyl carbanion equivalent (activated aldehyde) to a wide range of ketones. Few of these enzymes are known to accept such hindered and less active acceptor substrates, but intensive research has yielded a wide variety of products achievable through their catalysis.

This review aims to highlight ThDP‐dependent enzymes as the appropriate choice for the enzymatic synthesis of tertiary α‐hydroxy ketones and outline the latest findings. The summary given here will guide further development in the field of enzymatic tertiary alcohol formation by carboligation.

## 2 Ketone‐Accepting ThDP‐Dependent Enzyme

### 2.1 Decarboxylases

The decarboxylase (DC) superfamily is the largest among ThDP‐dependent enzymes^[^ ^44^ ^]^ and comprises 18 different subclasses, including pyruvate decarboxylase, benzaldehyde lyase,^[^ ^45^, ^46^ ^]^ acetohydroxyacid synthase (AHAS),^[^ ^47^ ^]^ pyruvate oxidase,^[^ ^48^, ^49^ ^]^ glyoxylate carboligase,^[^ ^50^, ^51^, ^52^ ^]^ and the PigD‐like proteins.^[^ ^37^, ^53^ ^]^ While the AHAS subclass is known to form tertiary alcohols using α‐keto esters as acceptors,^[^ ^47^ ^]^ the YerE‐like lyase is the only subclass of DC known to utilize isolated ketones in asymmetric carboligation reactions to form tertiary alcohols.

**Figure 1:**
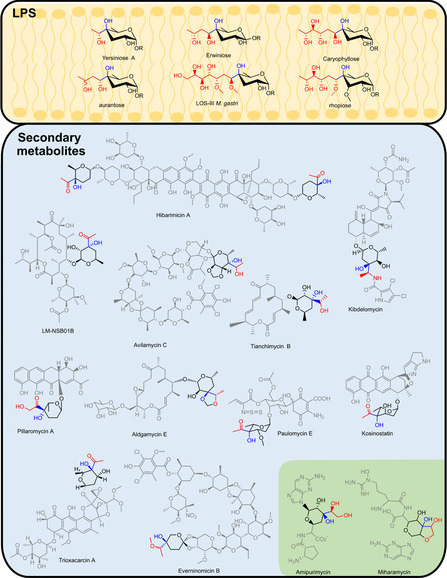
Structures of sugars, naturally branched by YerE‐like enzymes (yellow box) in the LPS, and αKADH2 (blue box) and TKs (green box) in secondary metabolites. The branches (or further modifications of it) introduced by ThDP‐dependent enzymes are in red. The resulting tertiary alcohol is highlighted in blue, the core structure of the branched sugar is in black, and the rest of the natural compound is in gray.

YerE from *Yersinia pseudotuberculosis* (*Yp*YerE) was the first DC to be characterized for aldehyde‐ketone cross‐coupling reactions. *Yp*YerE is physiologically involved in the formation of yersiniose A, a branched‐chain sugar found in the *O*‐antigen of the lipopolysaccharide (LPS) of the host and other bacteria (**Figure** **1**, yellow box).^[^ ^54^, ^55^, ^56^ ^]^ It catalyzes the decarboxylation of pyruvate and the transfer of activated acetaldehyde to cytidine diphosphate (CDP)‐3,6‐dideoxy‐4‐keto‐d‐glucose.^[^ ^57^ ^]^ The enzyme has a broad acceptor substrate tolerance including cyclic and open‐chain ketones as well as diketones and α‐ and β‐ketoesters with high to moderate (*R*) enantioselectivity (**Table** **1**).^[^ ^58^, ^59^ ^]^

**Table 1:** Reaction catalyzed by ThDP‐dependent enzymes for the synthesis of tertiary alcohols and range of products accessible *via* the different enzymes available.

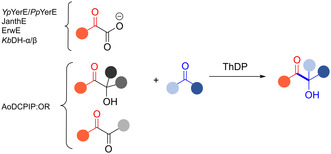			
Entry	Product	Conversion [*ee*]	Reference
1	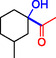	*Yp*YerE: 57 (*de* 99 *R*,*R* or *S*,*S*) *Kb*DH‐α/β: 57	[58, 98] [70]
2	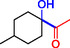	*Yp*YerE: 46/21 (isomers)	[58]
3	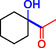	*Yp*YerE: 55 *Pp*YerE: 16 CDH_H28A_N484H: 18 *Kb*DH‐α/β: 90	[58, 59] [57, 72] [72] [70]
4	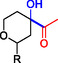	*R* = H *Yp*YerE: 57 PpYerE: 5 *Kb*DH‐α/β: 90 *R* = CH_3_ *Kb*DH‐α/β: 96	[58] [72] [70] [70]
5	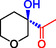	*Yp*YerE: 34 (84) CDH_H28A_N484H: 12 PpYerE: 37 (48) *Kb*DH‐α/β: 68 (63, R)	[58, 59] [72] [72] [70]
6	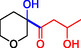	ErwE	[65]
7	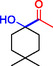	*Yp*YerE: 65	[58]
8	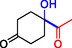	*Yp*YerE: 19	[58]
9	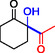	*Yp*YerE: 32 (22) CDH_H28A_N484H: 25 (88) Ao:DCPIP OR: 93 (69) *Kb*DH‐α/β: 14	[58, 59] [65] [88] [70]
10	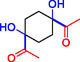	*Yp*YerE: 19/5 (diastereomers)	[58]
11	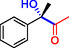	*Kb*DH‐α/β: 11 (99, *S*)	[67]
12	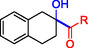	R = CH_3_ *Yp*YerE: 47 (9) R = (CH_2_)_2_CH_3_ JanthE: 3 (n.d.)	[58, 59] [62]
13	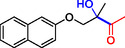	*Yp*YerE: 48 (91) AoDCPIP OR: 16 (61)	[59] [88]
14	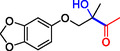	*Yp*YerE: 27 (87)	[58, 59]
15	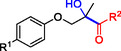	*R* ^2^ = CH_3_ *Yp*YerE: R^1^ = H: 48 (91) R^1^ = Cl: 45 R^1^ = NO_2_: 25 R^1^ = OH: 76 R^1^ = Br: 26 (63, *R*) *Pp*YerE: *R* ^1^ = H: 10 (93) CDH_H28A_N484H: *R* ^1^ = H: 55 (99) AoDCPIP OR: *R* ^1^ = H: 75 (85) *R* ^1^ = H JanthE: *R* ^2^ = CH_3_: 7 *R* ^2^ = CH_2_CH_3_: 20 (95, *R*) *R* ^2^ = (CH_2_)_2_CH_3_: 26 *R* ^2^ = CH_2_CH(CH_3_)_2_: 2	[58, 59] [57, 72] [72] [88] [62]
16	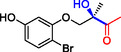	*Yp*YerE: 37 (78, *R*)	[58, 59]
17	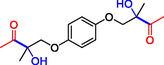	*Yp*YerE: 11	[58]
18	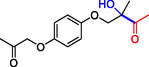	*Yp*YerE: 54	[58]
19	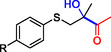	*Yp*YerE: *R* = H: 31 (3) *R* = Cl: 41 Ao:DCPIP OR: *R* = H: 63 (*rac*)	[58, 59] [88]
20	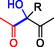	*R* = CH_3_ *Yp*YerE: 94 CDH_H28A_N484H: 31 Ao:DCPIP OR: 70 *R* = CH_2_CH_3_ Ao:DCPIP OR: 80 *R* = (CH_2_)_2_CH_3_ Ao:DCPIP OR: 35 *R* = (CH_2_)_3_CH_3_ Ao:DCPIP OR: 26 *R* = (CH_2_)_4_CH_3_ Ao:DCPIP OR: 30	[58] [72] [88] [88] [88] [88] [88]
21	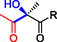	Ao:DCPIP OR *R* = CH_2_CH_3_: 61 (95, *R*) *R *= (CH_2_)_2_CH_3_: 53 (62, *R*) *R* = (CH_2_)_3_CH_3_: 53 (34, *R*) *R* = (CH_2_)_4_CH_3_: 68 (44, *R*) *R* = Ph: 45 (76, *R*)	[86, 91] [86, 91] [86, 91] [86, 99] [86, 91]
22	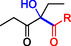	*R* = CH_3_ *Yp*YerE: 58 (84) CDH_H28A_N484H: 24 (98) *Pp*YerE: 66 (98) Ao:DCPIP OR: 100 (58, *R*) *R* = CH_2_CH_3_ Ao:DCPIP OR: 80 JanthE: *R* = CH_3_, CH_2_CH_3_, (CH_2_)_2_CH_3_, CH_2_CH(CH_3_)_2_: (n.d.)	[58, 59] [72] [57, 72] [88] [88] [62]
23	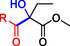	JanthE: *R* = CH_3_: 6 *R* = CH_2_CH_3_: 37 *R* = (CH_2_)_2_CH_3_: 56 *R* = CH_2_CH(CH_3_)_2_: 11	[62]
24	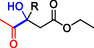	*Yp*YerE: *R* = CF_3_: 97 *R* = CH_3_: 42 (30)	[58, 59]
25	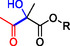	*R* = CH_3_ *Yp*YerE: >99 (30) CDH_H28A_H484A: 89 Ao:DCPIP OR: >99 (93) *R* = CH_2_CH_3_ Ao:DCPIP OR: 95 (85, *R*)	[58, 59] [72] [88] [100]
26	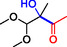	Ao:DCPIP OR: 90 (64)	[88]
27	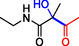	Ao:DCPIP OR: 40 (96)	[88]
28	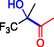	Ao:DCPIP OR: 100	[88]
29	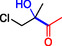	Ao:DCPIP OR: 57	[88]

Donor substrate tolerance is restricted to maximum C_3_‐chain transfer (pyruvate and 2‐oxobutyrate), with pyruvate being the preferred substrate. Since its first description, other enzymes with sequence similarity to *Yp*YerE have been characterized, and a pool of YerE‐like enzymes with unique substrate preferences is now available for the synthesis of various tertiary (*R*) alcohols.

ErwE from *Pectobacterium atrosepticum* catalyzes the C—C coupling of a hydroxy‐functionalized α‐keto acid with the deoxy keto sugar CDP‐4‐keto‐d‐3,6‐dideoxy‐glucose to form erwiniose (Figure 1, yellow box).^[^ ^60^, ^61^ ^]^ The reaction was demonstrated in vitro with a heterologously produced enzyme using a physiological substrate analogue, dihydro‐*2 H*‐pyran‐3(*4 H*)‐one (Table 1). Intriguingly, ErwE does not use pyruvate or acetaldehyde as donors but prefers chains longer than C_4_ and functionalized such as 4‐hydroxy‐2‐oxobutanoate, ‐pentanoate, or ‐hexanoate. JanthE is a novel YerE‐like enzyme with a unique ThDP‐binding motif, CDG instead of the canonical glycine aspartate glycine (GDG) amino acids, and with high substrate promiscuity for both donor and acceptor substrates. JanthE accepts short and longer donors (e.g., pyruvate, 2‐oxobutyrate, 2‐oxovalerate, 4‐methyl‐2‐oxopentanoate) and bulky cyclic and linear nonactivated ketones (e.g., 3,4‐hexanedione, phenoxy‐2‐propan‐2‐one, β‐tetralone) providing access to a wide range of (*R*)‐enantioenriched bulky tertiary alcohols (Table 1).^[^ ^62^ ^]^

Based on related natural product structures and the organization of the putative bacterial gene clusters (BGCs), other YerE‐like enzymes were predicted to catalyze similar branching reactions. MMAR_2332 from *Mycobacterium marinum* is known for the biosynthesis of caryophyllose, a highly branched and functionalized sugar found in the LPS of the microorganism (Figure 1, yellow box).^[^ ^63^, ^64^ ^]^ It has a similar donor substrate preference to ErwE. The enzyme has been biochemically characterized with regard to its activity with a hydroxy‐functionalized α‐keto acid and a benzaldehyde derivative.^[^ ^65^ ^]^ However, due to the instability of the purified enzyme, the investigation of its activity with a ketone as acceptor substrate is still pending.^[^ ^61^, ^63^, ^65^ ^]^ MygE is involved in the biosynthesis of gastriose, a branched sugar produced by *Mycobacterium gastri* (Figure 1, yellow box).^[^ ^66^ ^]^ MygE has a broad donor substrate promiscuity, ranging from C_3_ to C_8_ α‐keto acids when benzaldehyde is used as an acceptor. However, no activity with ketones was detected under the conditions tested in vitro.^[^ ^65^ ^]^ Other examples of C‐branched sugar structures occur in nature and have been characterized in the literature. For example, *Rhodopseudomonas palustris* and *Spirochaeta aurantia* produce functionalized C‐branched sugars, herein called ropiose (RPEPS‐30)^[^ ^67^ ^]^ and α‐aurantose (Figure 1, yellow box).^[^ ^68^, ^69^ ^]^ It is assumed that the putative ThDP‐dependent enzymes RhopE from *R. palustris* and SpirE from *S. aurantia* catalyze the C‐branching reactions; however, heterologous production did not result in ketone acceptance yet (unpublished results).

Recently, an *in silico* study based on BGC similarity identified more than 200 YerE‐like enzymes that are thought to catalyze cross‐coupling reactions with deoxy‐keto sugars.^[^ ^70^ ^]^ While for some of the identified genes a tertiary branched sugar structure is known from the literature, which could provide information about the physiological substrates of the newly identified ThDP‐dependent enzymes, for the vast majority a structure has not yet been identified.

Another member belonging to the DCs is cyclohexane‐1,2‐dione hydrolase (CDH) from *Azoarcus sp*. The enzyme physiologically catalyzes the C—C bond cleavage of cyclohexane‐1,2‐dione. Interestingly, the variant CDH_H28A_N484A can couple pyruvate with various ketones to form tertiary alcohols with an enantiomeric excess ranging from 54% to 94%.^[^ ^71^, ^72^ ^]^

Engineering studies to extend the substrate specificity or enantioselectivity of YerE‐like enzymes specifically for the synthesis of tertiary alcohols are not reported in the literature. The only attempt was limited at inverting the enantioselectivity of *Pp*YerE (a homologous of *Yp*YerE) for the synthesis of secondary alcohols. While the variants V479G and V479A showed promising results, further studies are needed to achieve complete enantioselectivity inversion.^[^ ^57^ ^]^ Furthermore, given the intrinsic chemical differences between ketones and aldehydes, the protein environment and residues governing the acceptance of the different substrates are expected to be different.

Overall, the DC superfamily is a valuable source of enzymes with specific donor and acceptor substrates preference that can be tailored to various needs. The many enzymes identified through bioinformatic work hold similar promising activity.

### 2.2 α‐Keto Acid Dehydrogenases 2

Several specialized natural products have tertiary α‐hydroxy ketones as a substructure. The ThDP‐dependent enzyme α‐keto acid dehydrogenase superfamily 2 (αKADH2) was proposed to introduce tertiary α‐hydroxy ketone substructure in different secondary metabolites (Figure 1, blue box). The first αKADH2 identified to catalyze such reaction is the one involved in the biosynthesis of aldgamycin E (more precisely in the biosynthesis of the sugar moiety D‐agarose), isolated in 1964^[^ ^73^ ^]^ (Figure 1, blue box). Just recently, the group of aldgamycins, containing the same acetyl branching was extended and now comprises more than 16 structures.^[^ ^74^ ^]^ Feeding and gene knockout experiments proved the activity of the αKADH2 using pyruvate as donor substrate.^[^ ^75^, ^76^ ^]^ Other specialized natural products are the quinocycline antibiotics kosinostatin (quinocyclin B)^[^ ^77^, ^78^, ^79^, ^80^ ^]^ and isoquinocycline A/B^[^ ^81^ ^]^ that were isolated from different bacteria and share the same branched sugar structure. The biosynthesis of kosinostatin has been proposed to include a αKADH2 similar to the one from aldgamycin.^[^ ^82^ ^]^ KbDH‐α/β, the αKADH2 enzyme from *Kibdelosporangium sp*. MA7385 is physiologically involved in the biosynthesis of the antibiotic kibdelomycin and its aldehyde–ketone cross‐coupling potential was investigated in vitro.^[^ ^70^ ^]^ In addition to being limited to pyruvate as donor substrate, conversion with several ketones, including the sugar analogues 2‐methyl‐tetrahydro‐4* H*‐pyran‐4‐one and dihydro‐2* H*‐pyran‐3(4* H*)‐one, acetophenone, and cyclohexanone was demonstrated. Interestingly, depending on the substrate, the enzyme is either (*R*) or (*S*) selective. For deoxy‐keto sugar analogues, the enzyme is (*R*) selective, while for nonphysiological substrates, such as acetophenone, it is (*S*) selective.

Other examples of natural products that contain a branched sugar and whose biosynthesis is presumably attributed to αKDH2 are listed in Figure 1. These all have a C_2_ branch (acetaldehyde equivalents as donor). Unlike the DC superfamily, where the branching sugars can have short to long branches, αKDH2 appears to be specialized for C_2_ branches.^[^ ^61^ ^]^ Nevertheless, the branches are differently modified, showcasing the great diversity that the introduced α‐hydroxy ketone precursor enables. The ketone of the branch is sometimes reduced (e.g., paulomycin F, trioxacarcin, avilamycin E), methylated (everinomycin B), oxidized (pillaromycin A),^[^ ^83^ ^]^ or transaminated (kibdelomycin). Furthermore, the branching either occurs in position C3 (e.g., aldgamycins, kibdelomycin) or C4 (e.g., trioxacarcin, paulomycin) leading to even more diversity (Figure 1, blue box).

Of all ketones‐branching ThDP‐dependent enzymes, the αKADH2 group is the one with the most known natural product structures. Currently, about 120 BGCs are known to contain a gene encoding an αKADH2 that catalyzes a similar reaction in the biosynthesis of potentially completely new structures.^[^ ^70^ ^]^

#### 2.2.1 Acetoin:dichlorophenolindophenol Oxidoreductase (Ao:DCPIP OR)

Unlike most of the enzymes described herein, the ThDP‐dependent acetoin:dichlorophenolindophenol oxidoreductase (Ao:DCPIP OR) is not known to synthesize branched sugars in nature. Ao:DCPIP OR is part of the acetoin dehydrogenase (AoDH enzyme system (ES)) of the genus *Bacillus* (among others),^[^ ^84^ ^]^ whose physiological role is to promote the oxidative cleavage of acetoin (2‐hydroxy‐3‐butanone). The resulting activated acetaldehyde is then transferred to the lipoamide cofactor of the second enzyme of the AoDH ES, which is responsible for the recycling of acetyl‐CoA.^[^ ^85^ ^]^ In 2010, Giovannini et al.^[^ ^86^ ^]^ discovered that Ao:DCPIP OR can catalyze the self‐condensation of diacetyl (2,3‐butanedione) to the tertiary α‐hydroxyketone acetyl‐acetoin (3‐hydroxy‐3‐methylpentane‐2,4‐dione). For this reason, it was originally named acetyl‐acetoin synthase.^[^ ^87^ ^]^ In recent years, the same authors have introduced the use of methyl acetoin (3‐hydroxy‐3‐methyl‐2‐butanone) as a nonphysiological acetyl anion donor which enables the enzyme to catalyze cross benzoin‐type condensations.^[^ ^88^ ^]^ By coupling this new donor with various activated ketones, an interesting array of enantioenriched chiral tertiary α‐hydroxyketones was obtained through aldehyde–ketone cross‐carboligation reaction (Table 1).

The donor substrate promiscuity of Ao:DCPIP OR is narrow as it can only transfer an *umpoled* acetaldehyde or propionaldehyde, with a clear preference for the acetyl group. On the acceptor side, a variety of methyl, ethyl, and cyclic activated ketones can be converted to the corresponding products with satisfactory yields and enantiomeric excesses (Table 1). Regarding ketone activation, substrates bearing ester, amide, keto, acetal, aromatic ether, and thioether groups have been successfully employed.^[^ ^88^ ^]^ In addition to (activated) ketones, Ao:DCPIP OR has also been shown to accept aromatic^[^ ^89^ ^]^ and aliphatic^[^ ^90^ ^]^ aldehydes, e.g., from propionaldehyde to biphenyl‐4‐carboxaldehyde. In terms of enantioselectivity, Ao:DCPIP OR exhibits consistent (*R*) selectivity in the synthesis of tertiary α‐hydroxy ketones.^[^ ^91^ ^]^ However, it is worth to note that in the cross‐coupling reactions with aromatic aldehydes, Ao:DCPIP OR yields the opposite enantiomer to that formed by most of the wild‐type ThDP‐enzymes. For instance, to date, the production of (*S*)‐phenylacetyl carbinol, an important intermediate in ephedrine synthesis, by wild‐type ThDP‐dependent enzymes only occurs with Ao:DCPIP OR.^[^ ^89^ ^]^

In the Thiamine diphosphate–dependent Enzymes Engineering Database (TEED),^[^ ^37^ ^]^ Ao:DCPIP OR is classified in the αKADH2 superfamily based on the enzymatic structure, even though the catalytic activity differs widely from the other enzymes of the same group. In a recent review,^[^ ^41^ ^]^ an exhaustive sequence similarity network search was conducted across all carboligases with documented biocatalytic activity. In this study, Ao:DCPIP OR showed no correlation with the other entries, emphasizing its distinct characteristics within the ThDP‐dependent enzyme families.

### 2.3 Transketolases

Transketolases (TKs) were proposed to catalyze the hydroxyacetyl transfer to a keto sugar in the biosynthesis of miharamycin and amipurimycin (Figure 1, green box).^[^ ^92^, ^93^, ^94^ ^]^ In a study by Zhang et al. several enzymes of the early steps of miharamycin biosynthesis have been characterized. However, the oxidoreductase that was proposed to introduce the keto function, and the subsequent branching by the split‐gene TK were not studied in vitro.^[^ ^95^ ^]^ Gene‐knockout experiments also failed to isolate the putative deoxy‐keto sugar substrate of the TK; instead, the substrate of the preceding oxidoreductase was found. Thus, the proposed function of TKs in the production of miharamycin and amipurimycin is still pending.^[^ ^92^ ^]^ Other TKs were also identified in the bioinformatic work of Krug et al.^[^ ^70^ ^]^ There are 13 putative sequences which, based on their BGC, supposedly catalyze a reaction with a ketone as an acceptor substrate. Interestingly, they all originate from *Streptomycetes* and are split‐gene TKs. None of these enzymes have yet been characterized in vitro and their use for tertiary alcohol synthesis has not been experimentally reported. Therefore, further research is needed to explore their capabilities for tertiary alcohols formation. The unique ability of TKs to use β‐hydroxy‐α‐keto acids as donor substrates combined with the potential use of ketones as acceptors would open the way to a new class of α‐functionalized tertiary alcohols susceptible to further modifications.

## 3 Summary and Outlook

The variety of possible substrate combinations, the high enantioselectivity, and the accessible starting materials make ThDP‐dependent enzymes attractive biocatalysts for synthesizing tertiary alcohols through C—C bond formation. Over the last 15 years, numerous enzymes with different substrate selectivity have been discovered, leading to the development of a versatile enzyme toolbox for a wide range of applications. Suitable enzymes can be selected from this toolbox depending on the desired product and starting materials. Many have a broad promiscuity for ketones as acceptor substrates, either activated (Ao:DCPIP OR) or nonactivated ones (YerE‐like and αKADH2), while the preference for donor substrate is more selective. YerE‐like enzymes use α‐keto acids of various lengths (C_2_ to C_8_), and KbDH‐α/β prefers pyruvate, while Ao:DCPIP OR is bound to methyl acetoin or diketones as donor substrates. Interestingly, mainly the (*R*)‐enantiomers of the tertiary alcohols are obtained enzymatically by wild‐type ThDP‐dependent enzymes. An exception is the (*S*)‐selective reaction catalyzed by KbDH‐α/β with acetophenone. Reversal of enantioselectivity should be possible, as it has been experimentally proven by engineering studies for the synthesis of acyloins of secondary alcohols.^[^ ^42^, ^96^ ^]^

The study of metabolic functions and natural product structures have revealed a wide variety of enzymatic pathways for the formation of α‐hydroxy ketones of tertiary alcohols and subsequent modifications (e.g., methylation, reduction, transamination, hydroxylation). These metabolites can serve as a source of inspiration to shed light on the great capabilities of ThDP‐dependent enzymes as catalysts for tertiary alcohols formation. While the substrate promiscuity of available enzymes is already impressive, for each superfamily, there are more than a hundred additional enzymes identified by BGC similarity that are likely to catalyze related reactions and could further expand the substrate promiscuity of this class of enzymes. TKs are some of the most promising enzymes. They have been proposed to accept ketones based on gene cluster analysis, but experimental evidence is still needed.

Research on ThDP‐dependent enzymes for the synthesis of tertiary alcohols has so far been at the discovery stage to identify novel enzymes and their wild‐type functions. The available enzymes and the associated substrate scope will serve as a starting point for future comparative and engineering studies. Expansion of both the donor and the acceptor substrate ranges and enantioselectivity should be pursued by tailoring each enzyme to specific needs. These studies will ideally lead to the development of biocatalysts capable of selectively utilizing a variety of donor and acceptor substrates and reaction conditions to obtain enantiopure products, similarly to what is now possible for the production of secondary alcohols with ThDP‐dependent enzymes.

Many other questions remain unanswered. Further studies should investigate the principal differences governing aldehyde versus ketone acceptance as well as the characteristics of substrate preferences. Although several structures of YerE‐like enzymes are available (*Pp*YerE and JanthE), co‐crystallized or soaked ketone substrates are needed to guide docking experiments. In addition, there is no experimentally determined structure of αKADH2 dehydrogenases, Ao:DCPIP OR, and TK enzymes that accept ketones. Given the diversity of domain organization among different superfamilies, structures elucidation by crystallization or Cryo‐EM may help to identify common features. Furthermore, molecular dynamics and quantum mechanics molecular modeling studies should be considered to determine the molecular mechanisms underlying the catalysis on ketones. There are very few studies on ThDP‐dependent enzyme dynamics, and most are limited to TK enzymes.^[^ ^97^ ^]^ Moreover, none have been applied to the production of tertiary alcohols. While the system is complex, such essential tools and methods applied to enzymes able to use ketones will help deepen our understanding of ThDP‐dependent catalysis.

Intriguingly, enzymes belonging to only three (i.e., DC, TK, αKADH2) out of nine superfamilies of ThDP‐dependent enzymes are known to utilize ketones as acceptor substrates. Given the same cofactor binding, it cannot be ruled out that enzymes from other superfamilies have similar potential. Each ThDP‐dependent enzyme superfamily has evolved for specific physiological roles in the formation of tertiary alcohols. YerE‐like enzymes are primarily involved in the biosynthesis of the host microorganism's cell structure such as the LPS. TK and αKADH2 are mainly involved in the biosynthesis of specialized natural products with antimicrobial activities, while Ao:DCPIP OR is involved in the acetoin catabolism. Similar metabolic pathways may exist in other microorganisms, and the use of gene cluster analysis and sequence similarity search tools may aid in the identification of novel promising enzymes. Surprising functions could be found in as yet‐unexplored orthologous enzymes in plants, viruses, or fungi. Likewise, other cellular structural compartments in microorganisms may have peculiar tertiary alcohol structures whose biosynthesis could be linked to new ThDP‐dependent enzymes. Nature holds a great diversity that will guide further exciting discoveries in this field.

## Conflict of Interest

The authors declare no conflict of interest.

## References

[1] W. W. Harding , Quaternary Stereocenters: Challenges And Solutions For Organic Synthesis (Eds: J. Christoffers , A. Baro ), Wiley-VCH, Wheinheim 2005.

[2] P. G. Cozzi , R. Hilgraf , N. Zimmermann , Eur. J. Org. Chem. 2007, 2007, 5969.

[3] F. Yang , J. Zhao , X. Tang , G. Zhou , W. Song , Q. Meng , Org. Lett. 2017, 19, 448. 28078895 10.1021/acs.orglett.6b03554

[4] B. Jung , M. S. Hong , S. H. Kang , Angew. Chem. Int. Ed. 2007, 46, 2616. 10.1002/anie.200604977 17330897

[5] K. B. Sharpless , W. Amberg , Y. L. Bennani , G. A. Crispino , J. Hartung , K. S. Jeong , H. L. Kwong , K. Morikawa , Z. M. Wang , J. Org. Chem. 1992, 57, 2768.

[6] H. Lebel , E. N. Jacobsen , Tetrahedron Lett. 1999, 40, 7303.

[7] E. Vedejs , M. Jure , Angew. Chem. Int. Ed. 2005, 44, 3974. 10.1002/anie.200460842 15942973

[8] Y. L. Liu , X. T. Lin , Adv. Synth. Catal. 2019, 361, 876.

[9] T. Desrues , X. Liu , J.-M. Pons , V. Monnier , J.-A. Amalian , L. Charles , A. Quintard , C. Bressy , Org. Lett. 2021, 23, 4332. 33999644 10.1021/acs.orglett.1c01261

[10] M. Hatano , K. Ishihara , Synthesis 2008, 2008, 1647.

[11] O. Riant , J. Hannedouche , Org. Biomol. Chem. 2007, 5, 873. 17340001 10.1039/b617746h

[12] J. L. Stymiest , V. Bagutski , R. M. French , V. K. Aggarwal , Nature 2008, 456, 778. 19079057 10.1038/nature07592

[13] M. Müller , ChemBioEng Rev. 2014, 1, 14.

[14] M. Bučko , K. Kaniaková , H. Hronská , P. Gemeiner , M. Rosenberg , Int. J. Mol. Sci. 2023, 24, 7334. 37108499 10.3390/ijms24087334 PMC10138715

[15] R. Ferraccioli , Symmetry 2021, 13, 1744.

[16] S. Kawanishi , K. Sugiyama , Y. Oki , T. Ikawa , S. Akai , Green Chem. 2017, 19, 411.

[17] S. Kawanishi , S. Oki , D. Kundu , S. Akai , Org. Lett. 2019, 21, 2978. 30875229 10.1021/acs.orglett.9b00334

[18] F. Kühn , S. Katsuragi , Y. Oki , C. Scholz , S. Akai , H. Gröger , Chem. Commun. 2020, 56, 2885. 10.1039/c9cc09103c 32037430

[19] L. G. Neto , C. D. F. Milagre , H. M. S. Milagre , Results Chem. 2023, 5, 100966.

[20] M. Engleder , H. Pichler , Appl. Microbiol. Biotechnol. 2018, 102, 5841. 29785499 10.1007/s00253-018-9065-7 PMC6013536

[21] I. Rottava , G. Toniazzo , P. F. Cortina , E. Martello , C. E. Grando , L. A. Lerin , H. Treichel , A. J. Mossi , D. de Oliveira , R. L. Cansian , O. A. C. Antunes , E. G. Oestreicher , LWT - Food Sci. Technol. 2010, 43, 1128.

[22] D. Brodkorb , M. Gottschall , R. Marmulla , F. Lüddeke , J. Harder , J. Biol. Chem. 2010, 285, 30436. 20663876 10.1074/jbc.M109.084244 PMC2945536

[23] G. A. Armstrong , M. Alberti , F. Leach , J. E. Hearst , Mol. Gen. Genet. 1989, 216, 254. 2747617 10.1007/BF00334364

[24] P. A. Scolnik , M. A. Walker , B. L. Marrs , J. Biol. Chem. 1980, 255, 2427. 7358679

[25] J. Jin , U. Hanefeld , Chem. Commun. 2011, 47, 2502. 10.1039/c0cc04153j 21243161

[26] Z. Sun , S. Shen , C. Wang , H. Wang , Y. Hu , J. Jiao , T. Ma , B. Tian , Y. Hua , Microbiology 2009, 155, 2775. 19443548 10.1099/mic.0.027623-0

[27] J. G. Zhu , J. T. Ochalek , M. Kaufmann , G. Jones , H. F. DeLuca , Proc. Natl. Acad. Sci. U. S. A. 2013, 110, 15650. 24019477 10.1073/pnas.1315006110 PMC3785760

[28] J. B. Cheng , M. A. Levine , N. H. Bell , D. J. Mangelsdorf , D. W. Russell , Proc. Natl. Acad. Sci. U. S. A 2004, 101, 7711. 15128933 10.1073/pnas.0402490101 PMC419671

[29] S. Lieberman , P. A. Warne , J. Steroid Biochem. Mol. Biol. 2001, 78, 299. 11717000 10.1016/s0960-0760(01)00105-4

[30] G. Weitnauer , A. Mühlenweg , A. Trefzer , D. Hoffmeister , R. D. Süßmuth , G. Jung , K. Welzel , A. Vente , U. Girreser , A. Bechthold , Chem. Biol. 2001, 8, 569. 11410376 10.1016/s1074-5521(01)00040-0

[31] M. Zhu , L. Wang , H. Zhang , L. Zhang , B. Tan , Q. Huang , Y. Zhu , C. Zhang , ACS Synth. Biol. 2023, 12, 1520. 37084337 10.1021/acssynbio.3c00055

[32] A. L. Lane , S.-J. Nam , T. Fukuda , K. Yamanaka , C. A. Kauffman , P. R. Jensen , W. Fenical , B. S. Moore , J. Am. Chem. Soc. 2013, 135, 4171. 23458364 10.1021/ja311065v PMC3611589

[33] V. Hélaine , C. Gastaldi , M. Lemaire , P. Clapés , C. Guérard-Hélaine , ACS Catal. 2022, 12, 733.

[34] V. Laurent , L. Gourbeyre , A. Uzel , V. Hélaine , L. Nauton , M. Traïkia , V. de Berardinis , M. Salanoubat , T. Gefflaut , M. Lemaire , C. Guérard-Hélaine , ACS Catal. 2020, 10, 2538.

[35] S. K. Bruffy , A. Meza , J. Soler , T. J. Doyon , S. H. Young , J. Lim , K. G. Huseth , P. H. Willoughby , M. Garcia-Borràs , A. R. Buller , Nat. Chem. 2024, 16, 2076. 39333392 10.1038/s41557-024-01647-1 PMC11611667

[36] V. Laurent , E. Darii , A. Aujon , M. Debacker , J. Petit , V. Hélaine , T. Liptaj , M. Breza , A. Mariage , L. Nauton , M. Traïkia , M. Salanoubat , M. Lemaire , C. Guérard-Hélaine , V. de Berardinis , Angew. Chem. Int. Ed. 2018, 57, 5467. 10.1002/anie.201712851 29542859

[37] M. Widmann , R. Radloff , J. Pleiss , BMC Biochem. 2010, 11, 9. 20122171 10.1186/1471-2091-11-9 PMC2831816

[38] S. Prajapati , F. Rabe von Pappenheim , K. Tittmann , Curr. Opin. Struct. Biol. 2022, 76, 102441. 35988322 10.1016/j.sbi.2022.102441

[39] F. Jordan , Nat. Prod. Rep. 2003, 20, 184. 12735696 10.1039/b111348h

[40] P. P. Giovannini , O. Bortolini , A. Massi , Eur. J. Org. Chem. 2016, 4441.

[41] H. Dobiašová , V. Jurkaš , P. Both , M. Winkler , ChemCatChem 2024, 16, 202301707.

[42] H. C. Hailes , D. Rother , M. Müller , R. Westphal , J. M. Ward , J. Pleiss , C. Vogel , M. Pohl , FEBS J. 2013, 280, 6374. 24034356 10.1111/febs.12496

[43] M. Müller , G. A. Sprenger , M. Pohl , Curr. Opin. Chem. Biol. 2013, 17, 261. 23523314 10.1016/j.cbpa.2013.02.017

[44] C. Vogel , J. Pleiss , Proteins 2014, 82, 2523. 24888727 10.1002/prot.24615

[45] Z. Ju , J. Xu , Z. Li , J. Fang , M. Li , D. C. Howell , F. E. Chen , Green. Synth. Catal. 2022, 3, 317.

[46] A. S. Demir , Ö. Şeşenoglu , E. Eren , B. Hosrik , M. Pohl , E. Janzen , D. Kolter , R. Feldmann , P. Dünkelmann , M. Müller , Adv. Synth. Catal. 2002, 344, 96.

[47] Y. Liu , Y. Li , X. Wang , Appl. Microbiol. Biotechnol. 2016, 100, 8633. 27576495 10.1007/s00253-016-7809-9

[48] K. Tittmann , R. Golbik , S. Ghisla , G. Hübner , Biochemistry 2000, 39, 10747. 10978159 10.1021/bi0004089

[49] Y. A. Muller , G. E. Schulz , Science 1993, 259, 965. 8438155 10.1126/science.8438155

[50] A. Kaplun , E. Binshtein , M. Vyazmensky , A. Steinmetz , Z. Barak , D. M. Chipman , K. Tittmann , B. Shaanan , Nat. Chem. Biol. 2008, 4, 113. 18176558 10.1038/nchembio.62

[51] N. Nemeria , E. Binshtein , H. Patel , A. Balakrishnan , I. Vered , B. Shaanan , Z. Barak , D. Chipman , F. Jordan , Biochemistry 2012, 51, 7940. 22970650 10.1021/bi300893v PMC3493109

[52] N. K. Gupta , B. Vennesland , Arch. Biochem. Biophys. 1966, 113, 255. 5328735 10.1016/0003-9861(66)90185-8

[53] C. Dresen , M. Richter , M. Pohl , S. Lüideke , M. Müller , Angew. Chem. Int. Ed. 2010, 49, 6600. 10.1002/anie.201000632 20669204

[54] R. P. Gorshkov , A. V. Zubkov , V. V. Isakov , Y. S. Ovodov , Carbohydr. Res. 1984, 126, 308. 6713436 10.1016/0008-6215(84)85389-6

[55] M. M. Cunneen , E. Pacinelli , W. C. Song , P. R. Reeves , Glycobiology 2011, 21, 1140. 21325338 10.1093/glycob/cwr010

[56] H. Chen , Z. Guo , H.-w. Liu , J. Am. Chem. Soc. 1998, 120, 11796.

[57] S. Hampel , J. P. Steitz , A. Baierl , P. Lehwald , L. Wiesli , M. Richter , A. Fries , M. Pohl , G. Schneider , D. Dobritzsch , M. Müller , ChemBioChem 2018, 19, 2283. 30101542 10.1002/cbic.201800325

[58] P. Lehwald , Biokatalytische Synthese tertiärer Alkohole mittels Asymmetrischer Carboligationsreaktion unter Verwendung eines Thiamindiphosphat-abhängigen Enzyms, Dissertation, University of Freiburg (DE) 2009.

[59] P. Lehwald , M. Richter , C. Röhr , H.-W. Liu , M. Müller , Angew. Chem. Int. Ed. 2010, 49, 2389. 10.1002/anie.200906181 PMC4170946 20191639

[60] S. N. Senchenkova , A. S. Shashkov , Y. A. Knirel , M. Ahmed , A. Mavridis , K. Rudolph , N. D. Zelinsky , Russ. Chem. Bull., Int. Ed. 2005, 54, 1276.

[61] J. P. Steitz , L. Krug , L. Walter , K. Hernández , C. Röhr , P. Clapés , M. Müller , Angew. Chem. Int. Ed. 2022, 61, e202113405. 10.1002/anie.202113405 PMC9306805 35092140

[62] L. Lanza , F. Rabe von Pappenheim , D. Bjarnesen , C. Leogrande , A. Paul , L. Krug , K. Tittmann , M. Müller , Angew. Chem. Int. Ed. 2024, 63, e202404045. 10.1002/anie.202404045 38874074

[63] Y. Rombouts , A. Burguière , E. Maes , B. Coddeville , E. Elass , Y. Guérardel , L. Kremer , J. Biol. Chem. 2009, 284, 20975. 19491094 10.1074/jbc.M109.011429 PMC2742863

[64] M. Adinolfi , M. Michela Corsaro , C. De Castro , A. Evidente , R. Lanzetta , P. Lavermicocca , M. Parrilli , Carbohydr. Res. 1996, 284, 119.

[65] J. P. M. Steitz , Characterisation of ThDP-Dependent Enzymes from the Biosynthesis of Longer-Branched Sugars, Dissertation, University of Freiburg (DE) 2019.

[66] M. Gilleron , J. Vercauterent , G. Puzo , J. Biol. Chem. 1993, 268, 3168. 8428994

[67] R. Marchetti , E. Bedini , D. Gully , R. Lanzetta , E. Giraud , A. Molinaro , A. Silipo , Org. Lett. 2018, 20, 3656. 29874087 10.1021/acs.orglett.8b01439

[68] C. J. Paul , E. A. Lyle , T. J. Beveridge , R. I. Tapping , A. M. Kropinski , E. Vinogradov , Glycoconjugate. J. 2009, 26, 1097. 10.1007/s10719-009-9230-4 19214746

[69] E. Vinogradov , C. J. Paul , J. Li , Y. Zhou , E. A. Lyle , R. I. Tapping , A. M. Kropinski , M. B. Perry , Eur. J. Biochem. 2004, 271, 4685. 15606756 10.1111/j.1432-1033.2004.04433.x

[70] L. Krug , D. Bjarnesen , L. Lanza , L. Lindemann , N. D. Fessner , M. Müller , Angew. Chem. Int. Ed. 2024, 63, e202403535. 10.1002/anie.202403535 38951114

[71] S. Loschonsky , T. Wacker , S. Waltzer , P. P. Giovannini , M. J. McLeish , S. L. A. Andrade , M. Müller , Angew. Chem. Int. Ed. 2014, 53, 14402. 10.1002/anie.201408287 25382418

[72] S. Loschonsky , Untersuchungen zur C-C-Bindungsknüpfungs- und C-C-Bindungsspaltungs-Aktivität des Thiamindiphosphat-abhängigen Enzyms Cyclohexan-1,2-Dion Hydrolase, Dissertation, University of Freiburg (DE) 2014.

[73] M. P. Kunstmann , L. A. Mitscher , E. L. Patterson , Antimicrob. Agents Chemother. 1964, 10, 87. 14288037

[74] K. Luo , Q. Yang , Y. Liu , C. Sun , S. Liu , J. Antibiot. 2024, 77, 842. 10.1038/s41429-024-00775-7 39322834

[75] R. Schmid , H. Grisebach , Eur. J. Biochem. 1970, 14, 243. 4319098 10.1111/j.1432-1033.1970.tb00283.x

[76] X. Tang , P. Dai , H. Gao , C. Wang , G. Chen , K. Hong , D. Hu , X. Yao , ChemBioChem 2016, 17, 1241. 27191535 10.1002/cbic.201600118

[77] T. Furumai , Y. Igarashi , H. Higuchi , N. Saito , T. Oki , J. Antibiot. 2002, 55, 128. 10.7164/antibiotics.55.128 12002993

[78] Y. Igarashi , H. Higuchi , T. Oki , T. Furumai , J. Antibiot. 2002, 55, 134. 10.7164/antibiotics.55.134 12002994

[79] W. D. Celmer , K. Murai , K. V. Rao , F. W. Tanner Jr , W. S. Marsh , Antibiot. Annu. 1957, 5, 484. 13521846

[80] T. J. McBride , A. R. English , Antibiot. Annu. 1957, 5, 493. 13521847

[81] M. Y. EI-Naggar , J. Microbiol. 2007, 45, 262.17618233

[82] H.-M. Ma , Q. Zhou , Y.-M. Tang , Z. Zhang , Y.-S. Chen , H.-Y. He , H.-X. Pan , M. C. Tang , J.-F. Gao , S.-Y. Zhao , Y. Igarashi , G.-L. Tang , Chem. Biol. 2013, 20, 796. 23790490 10.1016/j.chembiol.2013.04.013

[83] Z. Ji , Q. Nie , Y. Yin , M. Zhang , H. Pan , X. Hou , G. Tang , Angew. Chem. Int. Ed. 2019, 58, 18046. 10.1002/anie.201910882 31553109

[84] N. Krüger , F. B. Oppermann , H. Lorenzl , A. Steinbüchel , J. Bacteriol. 1994, 176, 3614. 8206840 10.1128/jb.176.12.3614-3630.1994 PMC205551

[85] F. B. Oppermann , B. Schmidt , A. Steinbüchel , J. Bacteriol. 1991, 173, 757. 1898934 10.1128/jb.173.2.757-767.1991 PMC207069

[86] P. P. Giovannini , P. Pedrini , V. Venturi , G. Fantin , A. Medici , J. Mol. Catal. B Enzym. 2010, 64, 113.

[87] S. Ui , K. Watanabe , T. Magaribuchi , Biosci. Biotechnol. Biochem. 1994, 58, 2271.

[88] G. Bernacchia , O. Bortolini , M. De Bastiani , L. A. Lerin , S. Loschonsky , A. Massi , M. Müller , P. P. Giovannini , Angew. Chem. Int. Ed. 2015, 54, 7171. 10.1002/anie.201502102 25914187

[89] P. P. Giovannini , L. A. Lerin , M. Müller , G. Bernacchia , M. De Bastiani , M. Catani , G. Di Carmine , A. Massi , Adv. Synth. Catal. 2016, 358, 2767.

[90] G. Di Carmine , O. Bortolini , A. Massi , M. Müller , G. Bernacchia , G. Fantin , D. Ragno , P. P. Giovannini , Adv. Synth. Catal. 2018, 360, 4132.

[91] P. P. Giovannini , G. Fantin , A. Massi , V. Venturi , P. Pedrini , Org. Biomol. Chem. 2011, 9, 8038. 22006343 10.1039/c1ob05928a

[92] F. Wang , W.-H. Zhang , J. Zhao , W.-J. Kang , S. Wang , B. Yu , H.-X. Pan , G.-L. Tang , J. Am. Chem. Soc. 2020, 142, 5996. 32167762 10.1021/jacs.0c01778

[93] A. J. Romo , T. Shiraishi , H. Ikeuchi , G.-M. Lin , Y. Geng , Y.-H. Lee , P. H. Liem , T. Ma , Y. Ogasawara , K. Shin-ya , M. Nishiyama , T. Kuzuyama , H. W. Liu , J. Am. Chem. Soc. 2019, 141, 14152. 31150226 10.1021/jacs.9b03021 PMC6774755

[94] A. J. Romo , Discovery of the Amipurimycin and Miharamycins Biosynthetic Gene Clusters and Insight into the Biosynthesis of Nogalamycin, PhD thesis, The University of Texas at Austin (US) 2016.

[95] W.-H. Zhang , F. Wang , Y.-L. Wang , S. You , H.-X. Pan , G.-L. Tang , Org. Lett. 2021, 23, 8761. 34747180 10.1021/acs.orglett.1c03254

[96] D. Gocke , L. Walter , E. Gauchenova , G. Kolter , M. Knoll , C. L. Berthold , G. Schneider , J. Pleiss , M. Müller , M. Pohl , ChemBioChem 2008, 9, 406. 18224647 10.1002/cbic.200700598

[97] L. Nauton , V. Hélaine , V. Théry , L. Hecquet , Biochemistry 2016, 55, 2144. 26998737 10.1021/acs.biochem.5b00787

[98] P. Lehwald , O. Fuchs , L. A. Nafie , M. Müller , S. Lüdeke , ChemBioChem 2016, 17, 1207. 27124802 10.1002/cbic.201600202

[99] O. Bortolini , P. P. Giovannini , S. Maietti , A. Massi , P. Pedrini , G. Sacchetti , V. Venturi , J. Mol. Catal. B Enzym. 2013, 85–86, 93.

[100] F. Presini , G. Di Carmine , P. P. Giovannini , V. Cristofori , L. A. Lerin , O. Bortolini , C. Trapella , A. Fantinati , Catalysts 2021, 11, 1440. The (*S*) absolute configuration was assigned in reference [88] based on reference data. Recent studies suggest that the right configuration is (*R*).

